# *Staphylococcus aureus* Phenol-Soluble Modulins α1–α3 Act as Novel Toll-Like Receptor (TLR) 4 Antagonists to Inhibit HMGB1/TLR4/NF-κB Signaling Pathway

**DOI:** 10.3389/fimmu.2018.00862

**Published:** 2018-04-25

**Authors:** Ming Chu, Mingya Zhou, Caihong Jiang, Xi Chen, Likai Guo, Mingbo Zhang, Zhengyun Chu, Yuedan Wang

**Affiliations:** ^1^Department of Immunology, School of Basic Medical Sciences, Peking University Health Science Center, Beijing, China; ^2^Key Laboratory of Medical Immunology, Ministry of Health, Peking University, Beijing, China; ^3^School of Food, Shihezi University, Shihezi, China; ^4^Pharmacy Departments, Liao Ning University of Traditional Chinese Medicine, Shenyang, China

**Keywords:** *Staphylococcus aureus*, phenol-soluble modulins, HMGB1, toll-like receptor 4, NF-κB, antagonists, inflammation, immune evasion

## Abstract

Phenol-soluble modulins (PSMs) have recently emerged as key virulence determinants, particularly in highly aggressive *Staphylococcus aureus* isolates. These peptides contribute to the pathogenesis of *S. aureus* infections, participating in multiple inflammatory responses. Here, we report a new role for *S. aureus* PSMs in high mobility group box-1 protein (HMGB1) induced inflammation by modulating toll-like receptor (TLR) 4 pathway. Direct ligation of TLR4 with *S. aureus* PSMα1–α3 and PSMβ1–β2 was identified by surface plasmon resonance. Remarkably, the binding affinity of TLR4 with HMGB1 was attenuated by PSMα1–α3. Further study revealed that PSMα1–α3 directly inhibited HMGB1-induced NF-κB activation and proinflammatory cytokines production *in vitro* using HEK-Blue hTLR4 cells and THP-1 cells. To analyze the molecular interactions between PSMs and TLR4, blast similarity search was performed and identified that PSMα1 and PSMβ2 were ideal templates for homology modeling. The three-dimensional structures of PSMα2, PSMα4, PSMβ1, and δ-toxin were successfully generated with MODELLER, and further refined using CHARMm. PSMs docking into TLR4 were done using ZDOCK, indicating that PSMα1–α3 compete with HMGB1 for interacting with the surrounding residues (336–477) of TLR4 domain. Our study reveals that *S. aureus* PSMα1–α3 can act as novel TLR4 antagonists, which account at least in part for the staphylococcal immune evasion. Modulation of this process will lead to new therapeutic strategies against *S. aureus* infections.

## Introduction

*Staphylococcus aureus* is one of the most common causes of human infections and death worldwide. When *S. aureus* first invades human body, there is a robust activation of multiple immune responses. To survive within the host, *S. aureus* has evolved a wide variety of virulence factors that interfere with the sophisticated immune defenses (1). Recently, a novel family of short, amphipathic, α-helical peptides found in staphylococci, coined as phenol-soluble modulins (PSMs), has attracted much attention owing to the key contribution to staphylococcal pathogenesis (2). PSMs were first isolated from *Staphylococcus epidermidis* culture filtrate by hot phenol extraction in 1999 with a description of “proinflammatory complex” (3). *S. aureus* PSMs were subsequently identified as a complex of seven PSMs, including PSMα1–α4, PSMβ1–β2, and δ-toxin, which have multiple roles in *S. aureus* infections (4–8). Ordered *S. aureus* PSMs aggregate into amyloid-like fibers can facilitate biofilm structuring, thereby protecting *S. aureus* from immune systems (9–13). While monomeric PSMs will disperse biofilms (14, 15). More importantly, PSMs can modulate immune response using aggregation as a control point for their activity (16).

The proinflammatory activity of *S. aureus* PSMs is arguably the most important contribution to staphylococcal pathogenesis (16). At nanomolar concentrations, *S. aureus* PSMs attract leukocytes and initiate immune responses *via* formyl-peptide receptor 2 (4, 17). While in the micromolar range, *S. aureus* PSMs can cause cytolysis of leukocytes after phagocytosis, leading to the release of damage-associated molecular patterns (DAMPs) (18–20). The best characterized DAMP is a nuclear protein, high mobility group box-1 protein (HMGB1), which directs the triggering of immune responses, bacterial killing, and tissue repair (21, 22). Toll-like receptor (TLR) 4 is required for HMGB1-induced inflammation (23). The interaction between HMGB1 and TLR4 promotes transcriptional activation of NF-κB and production of proinflammatory cytokines (24, 25). The HMGB1/TLR4 axis not only enables the immune system to sense an ongoing infection and recruit more immune cells but also initiates efficient host defenses to clear the pathogens. However, from work in recent years, *S. aureus* PSMs appear to have evolved to dampen the host defenses, enabling *S. aureus* to establish productive infections in the face of a robust immune response (26–30). Here, we gained insights into the action of *S. aureus* PSMs in the HMGB1/TLR4/NF-κB signaling pathway. This study will lead us to understand at least part of the underlying mechanisms of staphylococcal immune evasion.

## Materials and Methods

### Cell Culture

THP-1 cells were obtained from American Type Culture Collection (Manassas, VA, USA) and cultured in complete RPMI-1640 medium [10% fetal bovine serum (FBS), 2 mM l-glutamine, and 1% penicillin/streptomycin].

### HEK-Blue hTLR4 Cells

HEK-Blue hTLR4 cells and HEK-Blue Null2 cells (as control) were purchased from InvivoGen (San Diego, CA, USA). The HEK-Blue hTLR4 cells were obtained by co-transfection of the human TLR4 (hTLR4) gene, the myeloid differentiation factor 2 (MD-2) and CD14 co-receptor genes, and a secreted embryonic alkaline phosphatase (SEAP) reporter gene into HEK293 cells. The SEAP reporter gene is placed under the control of an IL-2 p40 minimal promoter fused to five NF-κB and AP-1 binding sites. Cells were grown in DMEM supplemented with 10% FBS, 2 mM l-glutamine, 100 µg/mL Normocin with selection antibiotic and passaged when 70% confluence was reached.

The activation of NF-κB can be monitored by a colorimetric assay quantifying the activity of the secreted SEAP in the cell supernatants in the presence of enzyme substrate as described by the manufacturer (InvivoGen).

### Surface Plasmon Resonance (SPR)

The recombinant hTLR4 protein was from R&D Systems (Minneapolis, MN, USA). The Catalog Number is 1478-TR. This recombinant hTLR4 protein consists of Glu24-Lys631 with a C-terminal Ser and 10-His tag, which is just the ectodomain with no transmembrane domain. The recombinant HMGB1 protein was provided by Kevin Tracey (The Feinstein Institute for Medical Research, Manhasset, NY, USA), which contains a disulfide bond between cysteines 23 and 45 and reduced thiol on cysteine 106, characterized by the liquid chromatography tandem mass spec-trometric analysis (23). This recombinant HMGB1 has been used as cytokine stimulator and confirmed to work for SPR analysis (23). The interaction of hTLR4 with HMGB1 and synthetic PSMs was analyzed by SPR spectroscopy with a Biacore T200 biosensor instrument (Biacore, Uppsala, Sweden). TLR4 was immobilized onto flow cells in a CM5 chip using an amine-coupling method. Binding analyses were carried out at 25°C and a flow rate of 30 µL/min. The disulfide HMGB1 and synthetic PSMs in 10 mM acetate buffer (pH = 5.2) was run over TLR4 at the gradient concentrations as indicated. An empty flow cell, without any immobilized protein, was used as a reference. For experiments using PSMs to block HMGB1–TLR4 interaction, HMGB1 was coated on the chip, hTLR4 was added as analyte (100 nM) plus increasing amounts of PSMs, and response were recorded. The binding curves were analyzed using a kinetic analysis supplied with the BIA evaluation software (Biacore) (11).

### Immunoprecipitation

The recombinant HMGB1 with a calmodulin-binding protein (CBP) tag was provided by Kevin Tracey (The Feinstein Institute for Medical Research, Manhasset, NY, USA) (23). The CBP-tagged HMGB1 or 10 µg CBP peptide alone was incubated overnight with 50 µL HEK-Blue hTLR4 cell lysates (precleared with calmodulin beads) at 4°C with gentle shaking. The mixture of HMGB1-CBP or CBP and HEK-Blue hTLR4 cell lysates was then incubated with 30 µL drained calmodulin beads for 1 h at 4°C. After extensive washing with PBS containing 0.1% Triton X-100, proteins bound to the beads were analyzed by immunoblotting with anti-TLR4 (R&D Systems) or anti-CBP antibodies.

### Stimulation Assays

Cells were stimulated with 1.0 µg/mL HMGB1 in the presence and absence of PSMs (PSMα1, 5 µg/mL; PSMα2, 5 µg/mL; PSMα3, 0.5 µg/mL, PSMα4, 5 µg/mL; PSMβ1, 10 µg/mL; PSMβ2, 10 µg/mL; δ-toxin, 2 µg/mL) (4). The involvement of the receptor for advanced glycation end products (RAGE) and TLR2 in HMGB1 signals in THP-1 cells was assessed using neutralizing antibodies against human RAGE (Chemicon, Temecula, CA, USA), TLR2 (eBioscience, San Diego, CA, USA) and TLR4 (eBioscience). Mouse IgG (eBioscience) was used as control. In the blocking experiments, THP-1 cells were preincubated with 20 µg/mL mouse IgG (as control) or neutralizing antibodies (20 µg/mL) against RAGE, TLR2, TLR4, or PSMα1 (5 µg/mL), PSMα2 (5 µg/mL), PSMα3 (0.5 µg/mL) for 30 min prior to stimulation with HMGB1 (1.0 µg/mL).

### Immunoblotting

Cells were collected in cold PBS, resuspended in hypotonic lysis buffer (10 mM HEPEs at pH 8.0, 1.5 mM MgCl_2_, 10 mM KCl, protease, and phosphatase inhibitors), and incubated on ice for 5 min. Cells were pelleted at 2,000 × *g* for 3 min. The cytoplasmic fractions were separated by SDS-PAGE and transferred onto a polyvinylidenedifluoride membrane (Amersham Biosciences, Little Chalfont, UK). Immunoblotting was performed using antibodies against phospho-NF-κB p65 (1:1,000, Cell Signaling, Boston, MA, USA) or NF-κB p65 (1:1,000, Cell Signaling) (31). β-actin (1:1,000, Santa Cruz, Dallas, TX, USA) and LaminB1 (1:1,000, Santa Cruz, Dallas, TX, USA) were used as controls (32).

### Luciferase Assay

THP-1 cells were transfected with a NF-κB-dependent luciferase reporter plasmid (Clontech, Mountain View, CA, USA) and stimulated as prior described. Cells were washed in PBS and lysed in Passive Lysis Buffer (Promega, Madison, WI, USA). The Dual-Luciferase reporter assay system (Promega) was used to quantitate both reporter genes by a POLARstar Omega multimode microplate spectrophotometer (BMG LABTECH) (31).

### Quantitative Real-Time PCR

Total RNA was recovered from cells using the Trizol reagent (Invitrogen, Waltham, MA, USA). RNA was reverse transcribed using the Superscript™ first-strand cDNA synthesis kit (Invitrogen). The primers were as follows: human TNF-α, sense 5′-ATGAGCACTGAAA GCATGATCC-3′ and antisense 5′-GAG GGC TGA TTA GAG AGA GGT C-3′; human IL-6, sense 5′-CCA GCT ATG AAC TCC TTC TC-3′ and antisense 5′-GCT TGT TCC TCA CAT CTC TC-3′; and GAPDH, sense 5′-ACC CAC TCC TCC ACC TTT GA-3′ and antisense 5′-CTG TTG CTG TAG CCA AAT TCG T-3′. The relative expression was calculated using 2^−ΔΔCt^ method (33).

### Cytokine Analysis

The concentration of TNF-α and IL-6 in the cell supernatants were determined using commercially obtained enzyme-linked immunosorbent assay (ELISA) kits according to the manufacturer’s instructions (R&D Systems) (34).

### Homology Modeling

Homology modeling was carried out using the software package Discovery Studio 2017R2 (Accelrys, San Diego, CA, USA). The structure of PSMα1 (PDB ID: 5KHB) was found *via* BLAST and used as a template in the alignment and modeling of PSMα2 and PSMα4. PSMβ2 (PDB ID: 5KGZ) was selected for PSMβ1 modeling (35). And the three-dimensional (3D) structure of deta-toxin (PDB ID: 2KAM) was an ideal template for *S. aureus* δ-toxin. Homology model was constructed using MODELLER.

### Receptor–Ligand Interaction

ZDOCK was applied in the docking of HMGB1 (PDB ID: 2LY4) and all the *S. aureus* PSMs to TLR4 (PDB ID: 3FXI) (36, 37). Angular step size for the rotational sampling of the ligand orientations was set to 6, and a total of 3,000 poses were generated for each ligand-receptor complex configuration. All the generated docking poses were further optimized using RDOCK program for CHARMm force field refinement, and the best-score and lowest-energy models were selected. The model complex structures located on the dimerization interface of TLR4/MD-2 were excluded from the remaining poses within 100 by referring to LPS bound form of the TLR4/MD-2 dimer (PDB ID: 3FXI) (37). The protein interaction energy was calculated by using the sum of electrostatic and van de Waals interaction terms (33). All the selected poses of TLR4 with HMGB1 or *S. aureus* PSMs were subjected to 10 ns molecular dynamics (MD) simulations using Discovery Studio 2017R2. The stability of the complex was analyzed and confirmed by plotting root mean square deviation (RMSD). The RMSD is a measure of the deviation of the conformational stability of the proteins from backbone structure to the early starting structure and fundamental property investigation in MD studies.

### Statistical Analysis

The results were conducted using Student’s *t*-test with SPSS 13.0 software. The data were expressed as mean ± SEM of three independent experiments. Values of *p* < 0.001 were considered to be statistically significant (38).

## Results

### *S. aureus* PSMs α1–α3 and β1–β2 Bind to hTLR4

**Figure 1:**
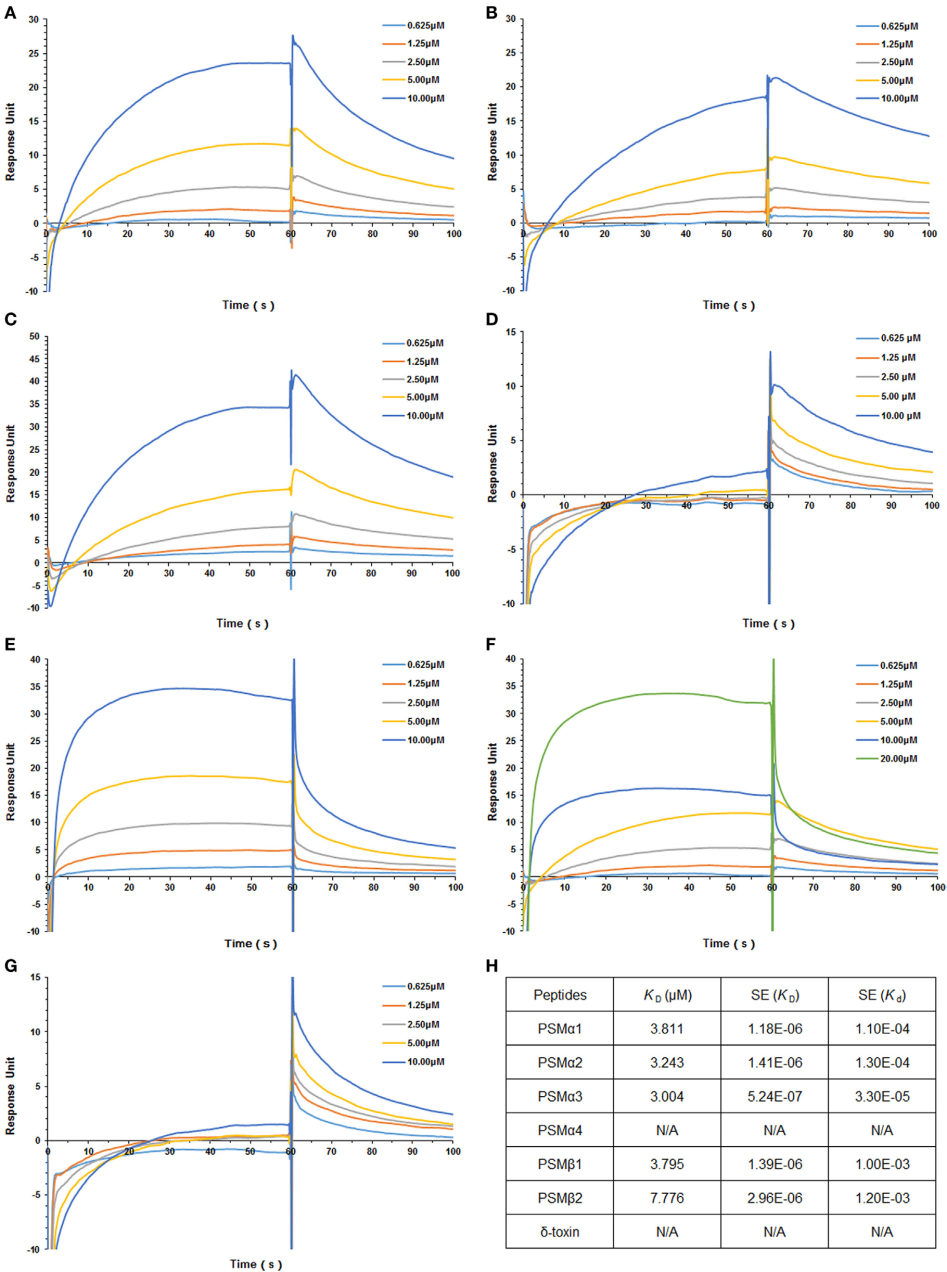
*Staphylococcus aureus* phenol-soluble modulins (PSMs) α1–α3 and β1–β2 bind to human TLR4 (hTLR4). Surface plasmon resonance analysis was performed to assess *S. aureus* PSMs binding to hTLR4 (coated on the chip), including PSMα1 **(A)**, PSMα2 **(B)**, PSMα3 **(C)**, PSMα4 **(D)**, PSMβ1 **(E)**, PSMβ2 **(F)**, and δ-toxin **(G)**. *S. aureus* PSMs binding to toll-like receptor (TLR) 4 was tested at different concentrations (0.625, 1.25, 2.5, 5.0, and 10.0 µM). **(H)** The binding affinity of TLR4 with *S. aureus* PSMs. *K*_d_ represents the dissociation constant and *K*_D_ represents the equilibrium dissociation constant. *K*_D_ (μM), SE (*K*_D_), and SE (*K*_d_) were shown. Data are presented as response units over time (seconds) and are representative of three experiments.

*Staphylococcus aureus* PSMs and TLR4 binding was analyzed by SPR, in which *K*_d_ represents the dissociation constant and *K*_D_ represents the equilibrium dissociation constant. TLR4 was coated on the sensor chip and then probed with the synthetic *S. aureus* PSMs, including PSMα1–α4, PSMβ1–β2, and δ-toxin. We identified significant PSMα1–α3 and PSMβ1-β2 binding to TLR4 in a concentration-dependent manner, with apparent *K*_D_ of 3.811, 3.243, 3.004, 3.795, and 7.776 µM, respectively, in which the error ranges for *K*_d_ and *K*_D_ were shown (Figure 1; Figures S1A–E in Supplementary Material). However, PSMα4 and δ-toxin do not bind to TLR4, indicating that PSMα1–α3 and PSMβ1–β2 binding to TLR4 is specific. Consequently, PSMα4 and δ-toxin were used as negative controls in the further study. In addition, we analyzed the binding activity of all the synthetic *S. aureus* PSMs with recombinant human CD14 and MD-2 protein by SPR. As a result, all these PSMs do not bind to CD14 or MD-2 (data not shown), indicating that PSMs α1–α3 and β1–β2 can bind to hTLR4 directly.

### *S. aureus* PSMs α1–α3 Attenuate HMGB1–TLR4 Binding

**Figure 2:**
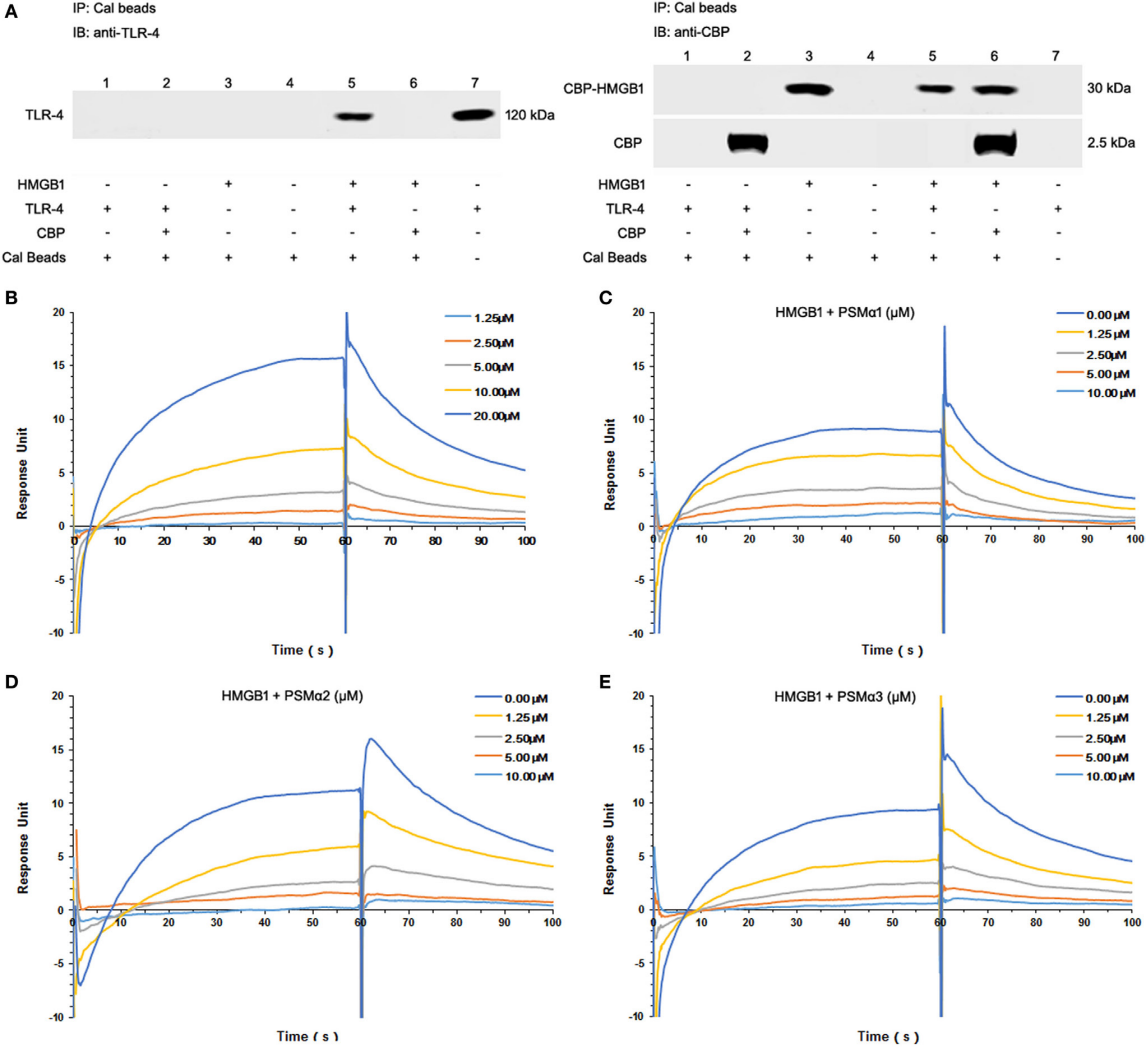
*Staphylococcus aureus* phenol-soluble modulins (PSMs) α1–α3 attenuate HMGB1-toll-like receptor (TLR4) binding. **(A)** Mixture of calmodulin-binding protein (CBP)-tagged HMGB1 or CBP alone with the HEK-Blue hTLR4 cell lysates was immunoprecipitated (IP) with calmodulin beads, and immunoblotted (IB) with anti-human TLR4 (hTLR4) or CBP antibodies. Recombinant hTLR4 protein was included as positive control (right lane 7). **(B)** Surface plasmon resonance (SPR) analysis was performed to assess HMGB1 binding to hTLR4. HMGB1 binding to hTLR4 was tested at different concentrations (1.25, 2.5, 5.0, 10.0, and 20 µM) with a *K*_D_ value of 9.199 µM. SPR analysis of HMGB1 binding to TLR4 was performed in the presence of PSMα1 **(C)**, PSMα2 **(D)**, PSMα3 **(E)** as shown. Data were representative of three repeats.

To study HMGB1–TLR4 interactions, immunoprecipitation were used to pull down TLR4 from HEK-Blue hTLR4 cell lysates. Coincubation with CBP-tagged HMGB1, but not CBP tag alone, pulled down hTLR4 from TLR4-expressing HEK-Blue hTLR4 cell lysates, confirming that HMGB1 effectively binds to TLR4 (Figure 2A).

Moreover, we observed significant HMGB1 binding to TLR4 in a concentration-dependent manner, with an apparent *K*_D_ of 9.199 µM (Figure 2B; Figure S1F in Supplementary Material). Having identified all the synthetic *S. aureus* PSMs failed to bind to HMGB1 (data not shown), HMGB1 was coated on the chip, TLR4 was added as analyte (100 nM) plus increasing amounts of PSMs. Remarkably, PSMα1–α3 attenuated HMGB1–TLR4 binding in a concentration-dependent manner, indicating that *S. aureus* PSMα1–α3 might affect HMGB-induced TLR4 activation (Figures 2C–E).

### *S. aureus* PSMs α1–α3 Inhibit HMGB1-Induced NF-κB Activation

**Figure 3:**
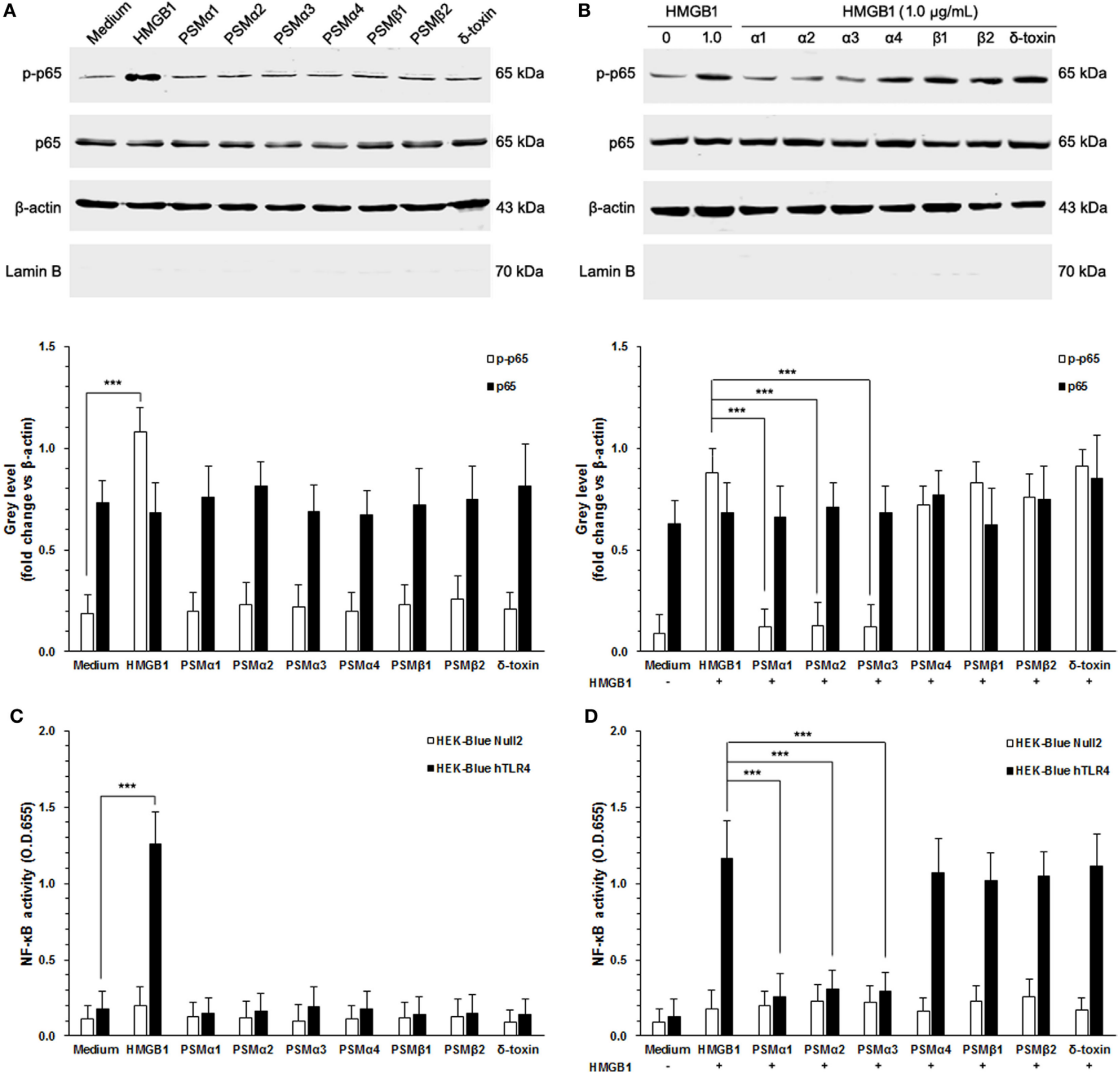
*Staphylococcus aureus* phenol-soluble modulins (PSMs) α1–α3 inhibit HMGB1-induced NF-κB activation. **(A)** HEK-Blue hTLR4 cells were stimulated for 30 min with 1.0 µg/mL recombinant HMGB1 or *S. aureus* PSMs (PSMα1, 5 µg/mL; PSMα2, 5 µg/mL; PSMα3, 0.5 µg/mL, PSMα4, 5 µg/mL; PSMβ1, 10 µg/mL; PSMβ2, 10 µg/mL; δ-toxin, 2 µg/mL). NF-κB p65 protein and phosphorated p65 at Ser536 in the cytoplasm were assessed by immunoblotting. β-actin and LaminB were used as control. LaminB in the cytoplasm was detected to ensure that there was no contamination during the fractionation. **(B)** Phosphorylation of NF-κB in HEK-Blue hTLR4 cells challenged with HMGB1 in the presence and absence of *S. aureus* PSMs as prior described. **(C)** HEK-Blue hTLR4 cells and HEK-Blue Null2 cells (as control) were stimulated for 30 min with 1.0 µg/mL recombinant HMGB1 or *S. aureus* PSMs as prior described. NF-κB-induced secreted embryonic alkaline phosphatase (SEAP) activity was assessed using QUANTI-Blue and by reading the OD at 655 nm. **(D)** The NF-κB-induced SEAP activity of HEK-Blue hTLR4 cells or HEK-Blue Null2 cells (as control) challenged with HMGB1 in the presence and absence of *S. aureus* PSMs as prior described.

It has been appreciated that TLR4 is required for HMGB1-mediated proinflammatory responses (23). To evaluate HMGB1 signals *via* TLR4, we chose HEK-Blue hTLR4 cells obtained by co-transfection of the hTLR4, MD-2, and CD14 co-receptor genes, and an inducible SEAP reporter gene into HEK293 cells. HEK-Blue Null2 cells that lack endogenous HMGB1 receptors, which are HMGB1-unresponsive, were used as control. As shown in Figure 3A, HEK-Blue hTLR4 cells showed high HMBG1 sensitivity, thereby phosphorylation of NF-κB, whereas did not respond to the *S. aureus* PSMs (Figure 3A). In addition, neither HMGB1 nor *S. aureus* PSMs increased the expression of endogenous NF-κB in HEK-Blue hTLR4 cells (Figure 3A). It is remarkable that *S. aureus* PSMs α1–α3 significantly inhibited HMGB1-mediated phosphorylation of NF-κB, whereas PSMα4, PSMβ1, PSMβ2, and δ-toxin did not (Figure 3B). Furthermore, the activation of NF-κB was detected by a colorimetric assay quantifying the activity of the secreted SEAP in the supernatants of HEK-Blue hTLR4 cells. As a result, HMGB1-induced NF-κB activation within 6 h was significantly inhibited by *S. aureus* PSMs α1–α3 (Figures 3C,D). These results demonstrated that *S. aureus* PSMs α1–α3 inhibit HMGB1/TLR4/NF-κB signaling.

### *S. aureus* PSMs α1–α3 Suppress HMGB1-Induced Inflammatory Responses

**Figure 4:**
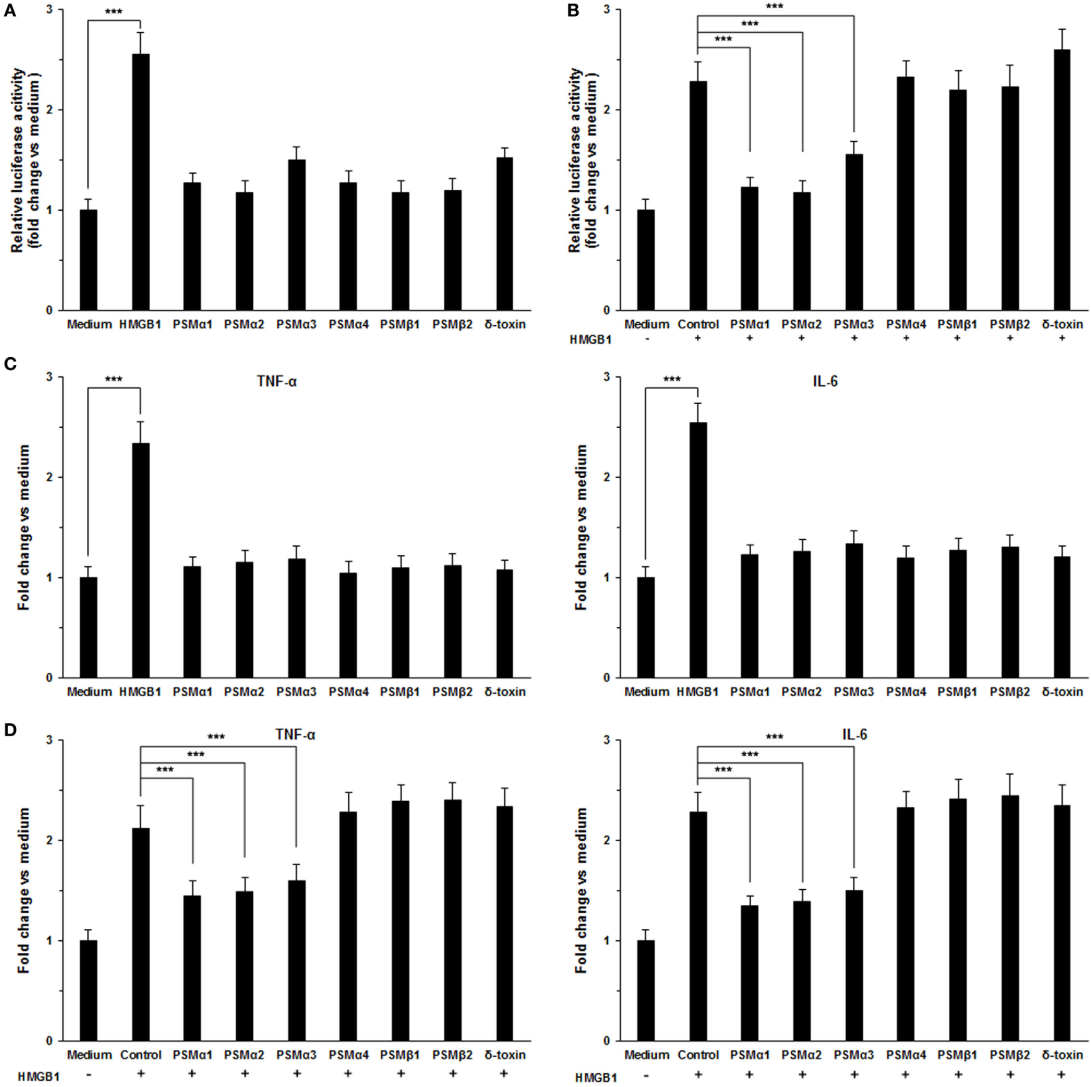

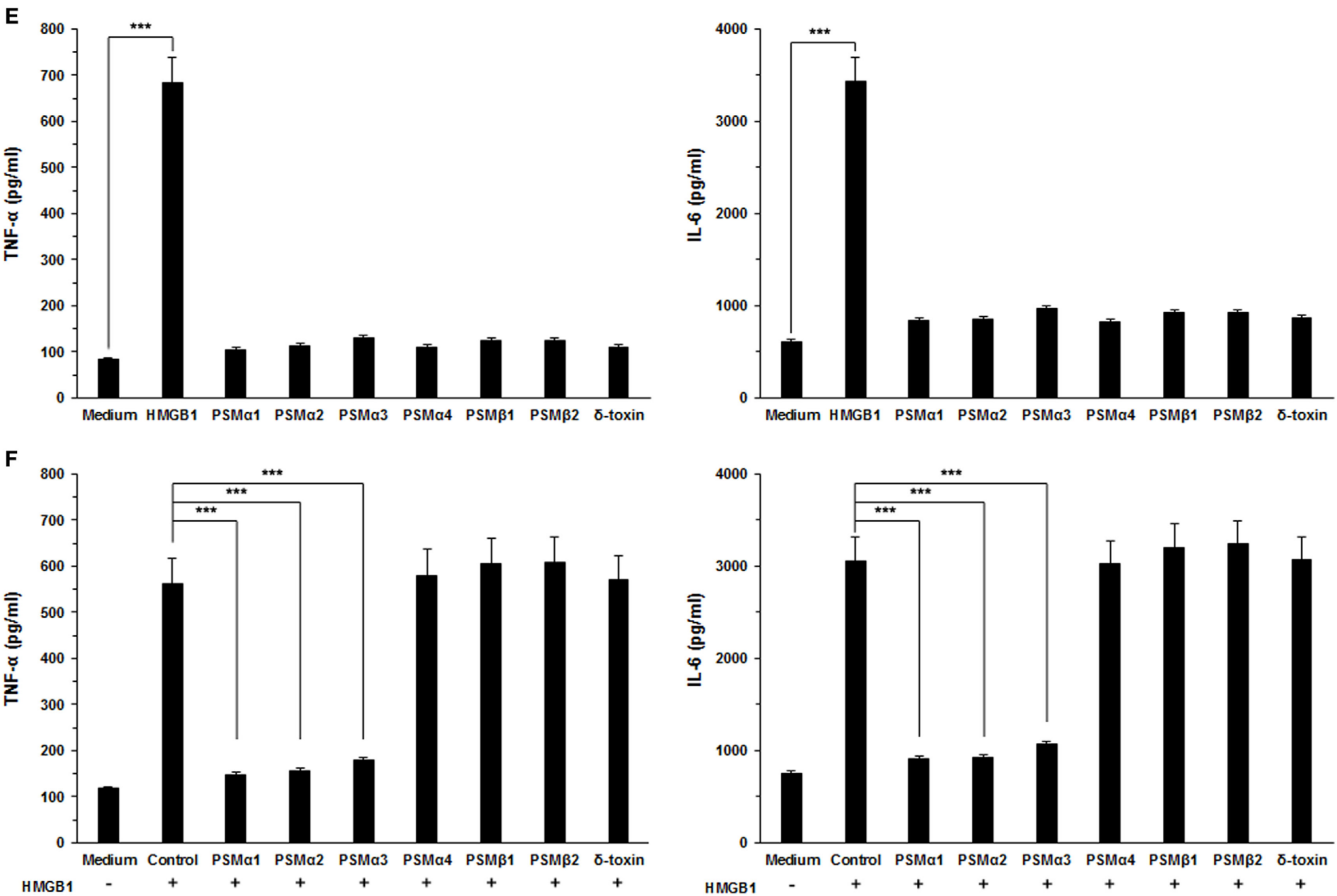
*Staphylococcus aureus* phenol-soluble modulins (PSMs) α1–α3 inhibit HMGB1-induced inflammatory response. **(A)** THP-1 cells were transfected with a NF-κB-dependent luciferase reporter. The luciferase activity of THP-1 cells was measured after stimulated with 1.0 µg/mL recombinant HMGB1 or *S. aureus* PSMs (PSMα1, 5 µg/mL; PSMα2, 5 µg/mL; PSMα3, 0.5 µg/mL, PSMα4, 5 µg/mL; PSMβ1, 10 µg/mL; PSMβ2, 10 µg/mL; δ-toxin, 2 µg/mL). **(B)** The NF-κB activity of THP-1 cells challenged with HMGB1 in the presence and absence of *S. aureus* PSMs as prior described. The values for medium treated cells were arbitrarily expressed as 1.0. **(C)** THP-1 cells were stimulated for 12 h with 1.0 µg/mL recombinant HMGB1 or *S. aureus* PSMs as prior described. The expression of human TNF-α and IL-6 was assessed by RT-PCR. **(D)** The expression of human TNF-α and IL-6 in THP-1 cells challenged with HMGB1 in the presence and absence of *S. aureus* PSMs as prior described. **(E)** THP-1 cells were stimulated with 1.0 µg/mL recombinant HMGB1 or *S. aureus* PSMs as prior described for 24 h. Supernatants were analyzed for human TNF-α and IL-6 by enzyme-linked immunosorbent assay. **(F)** The levels of human TNF-α and IL-6 in the supernatants of THP-1 cells challenged with HMGB1 in the presence and absence of *S. aureus* PSMs. Data shown are mean ± SEM (*n* = 5). Significance was calculated by Student’s *t*-test. ***p < 0.001.

To validate the role of *S. aureus* PSMs α1–α3 on HMGB1-induced inflammatory responses, we measured the NF-κB activity and proinflammatory cytokines production in THP-1 cells, including TNF-α and IL-6. We observed that HMGB1-induced NF-κB acti-vation within 6 h was significantly inhibited by *S. aureus* PSMs α1–α3, but not by PSMα4, PSMβ1–β2, and δ-toxin (Figures 4A,B). HMGB1 challenge resulted in a significant increase in the expression of TNF-α and IL-6 after 12 h of stimulation, whereas no increase was observed in the presence of the *S. aureus* PSMs (Figure 4C). Notably, the expression of HMGB1-induced TNF-α and IL-6 was significantly inhibited by PSMs α1–α3 (Figure 4D). In addition, we measured the levels of TNF-α and IL-6 in the supernatants. Consequently, TNF-α and IL-6 release into the supernatants 24 h after treatment with the combination of HMGB1 and *S. aureus* PSMs α1–α3 were significantly lower than following treatment with HMGB1 alone (Figures 4E,F). To examine the involvement of RAGE and TLR2 in HMGB1 signals in THP-1 cells, we performed a blocking experiment. As shown in Figure S2 in Supplementary Material, HMGB1-mediated TNF-α and IL-6 release was inhibited by anti-TLR4 antibodies, but not by anti-TLR2 or RAGE antibodies (25). More importantly, HMGB1-induced TNF-α and IL-6 release in the THP-1 cells which were preincubated with *S. aureus* PSMα1–α3 also decreased significantly, indicating that PSMα1–α3 have the same function as neutralizing antibody against hTLR4 by specifically targeting HMGB1–TLR4 interactions (Figure S2 in Supplementary Material).

### Homology Modeling of *S. aureus* PSMs

**Figure 5:**
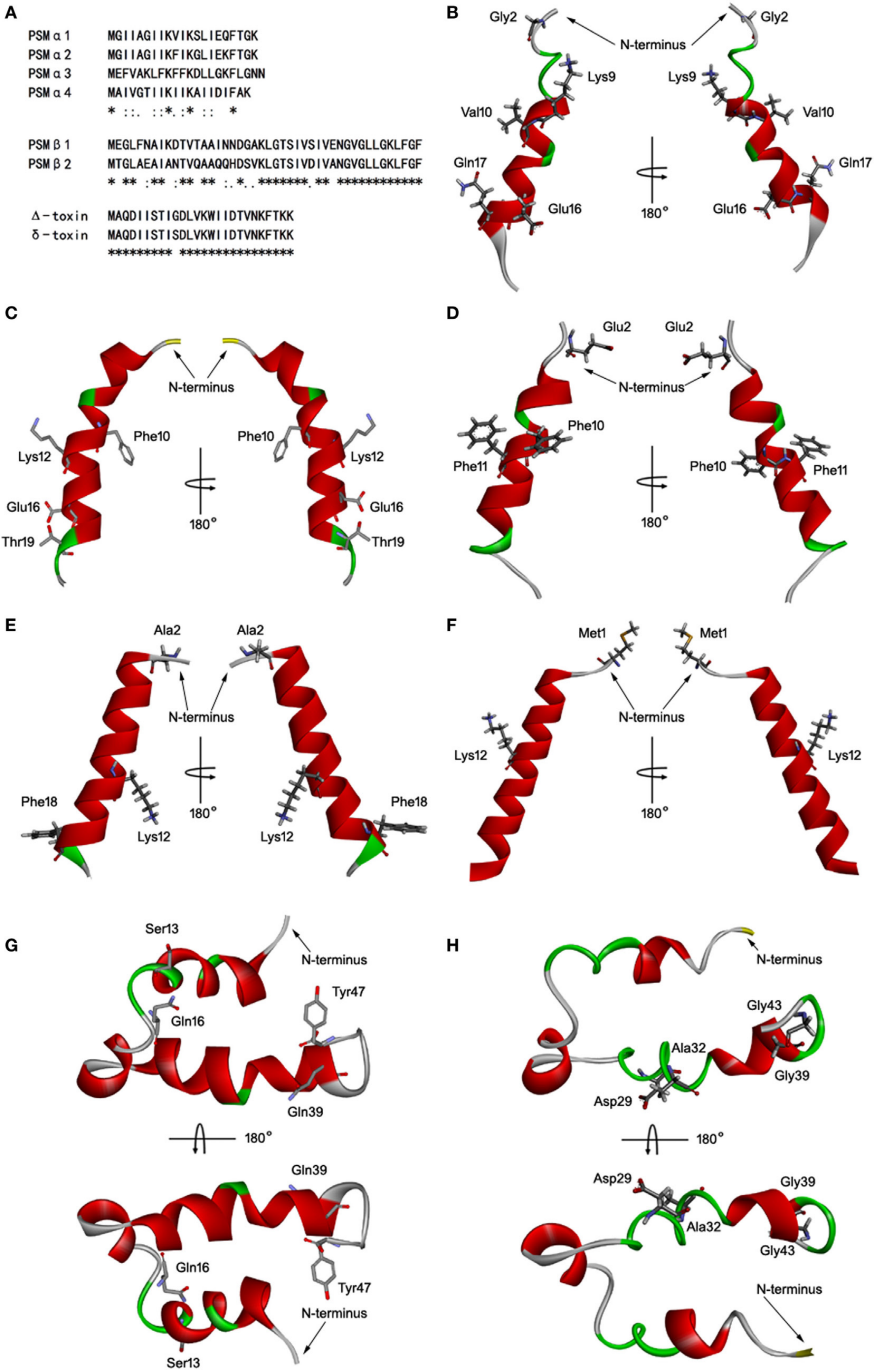
Homology modeling of *Staphylococcus aureus* phenol-soluble modulins (PSMs). **(A)** Alignment of amino acid sequences of PSMα1, PSMα2, PSMα3, PSMα4, PSMβ1, PSMβ2, and δ-toxin. Sequence alignments were generated using Clustal Omega. Conserved, conservative, and semiconservative substitutions are indicated using asterisks, colons, and periods, respectively. The three-dimensional model of PSMα1 **(B)**, PSMα2 **(C)**, PSMα3 **(D)**, PSMα4 **(E)**, δ-toxin **(F)**, PSMβ1 **(G)**, and PSMβ2 **(H)** were generated by MODELLER. Side chains that may participate in salt bridges are shown explicitly. Secondary structural elements are depicted as ribbons (coils, α-helices; arrows, β-sheets). Color is based on secondary structures (α-helices, red; β-sheets, sky blue; loops, green). The side chains are shown as sticks with carbon, oxygen, and nitrogen colored gray, red, and blue.

*Staphylococcus aureus* PSMs are a family of amphipathic pep-tides, including PSMα1–α4, PSMβ1–β2, and δ-toxin. Determination of the 3D structures, in combination with the data already acquired for these peptides, is essential for detailed understanding of their biological function. Fortunately, the 3D structures of PSMα1, PSMα3, and PSMβ2 has been elucidated using nuclear magnetic resonance spectroscopy (NMR) (35). Blast similarity search in DS modeling was performed and identified PSMα1 and PSMβ2 were ideal templates for homology modeling of PSMα2, PSMα4, and PSMβ1. PSMα2 and PSMα4 share more sequence homology with PSMα1 than with PSMα3 (Figure 5A). PSMβ1 and PSMβ2 share a high degree of sequence homology, but PSMβ2 exhibits an overall neutral charge whereas PSMβ1 is slightly anionic (Figure 5A). In addition, the 3D structure of delta-toxin in 2KAM (Δ-toxin) was found *via* BLAST, which shares 96% sequence homology with the *S. aureus* δ-toxin (Figure 5A). The 10th amino acid in the δ-toxin is Ser, whereas in the Δ-toxin is Gly (4). The 3D model of PSMα2, PSMα4, PSMβ1, and δ-toxin were generated with MODELLER program. Structurally, PSMα2 and PSMα4 are similar to PSMα1. Each contains a single α-helix with a slight bend in it (Figures 5B–E). Like α-type PSM, δ-toxin also contains one α-helix (Figure 5F). PSMβ1 contains three amphipathic helices similar to PSMβ2 (Figures 5G,H). The first begins at residue 2 and continues to residue 16. This helix has a slight bend and interacts with the third helix. The second helix is the shortest, runs from residue 18 to 23. Finally, the third and longest helix runs from residue 24 to 40. The interface between α-helices 1 and 3 is such that hydrophobic residues interact, forming a hydrophobic core.

### *S. aureus* PSMs α1–α3 Disrupt HMGB1–TLR4 Interactions

**Figure 6:**
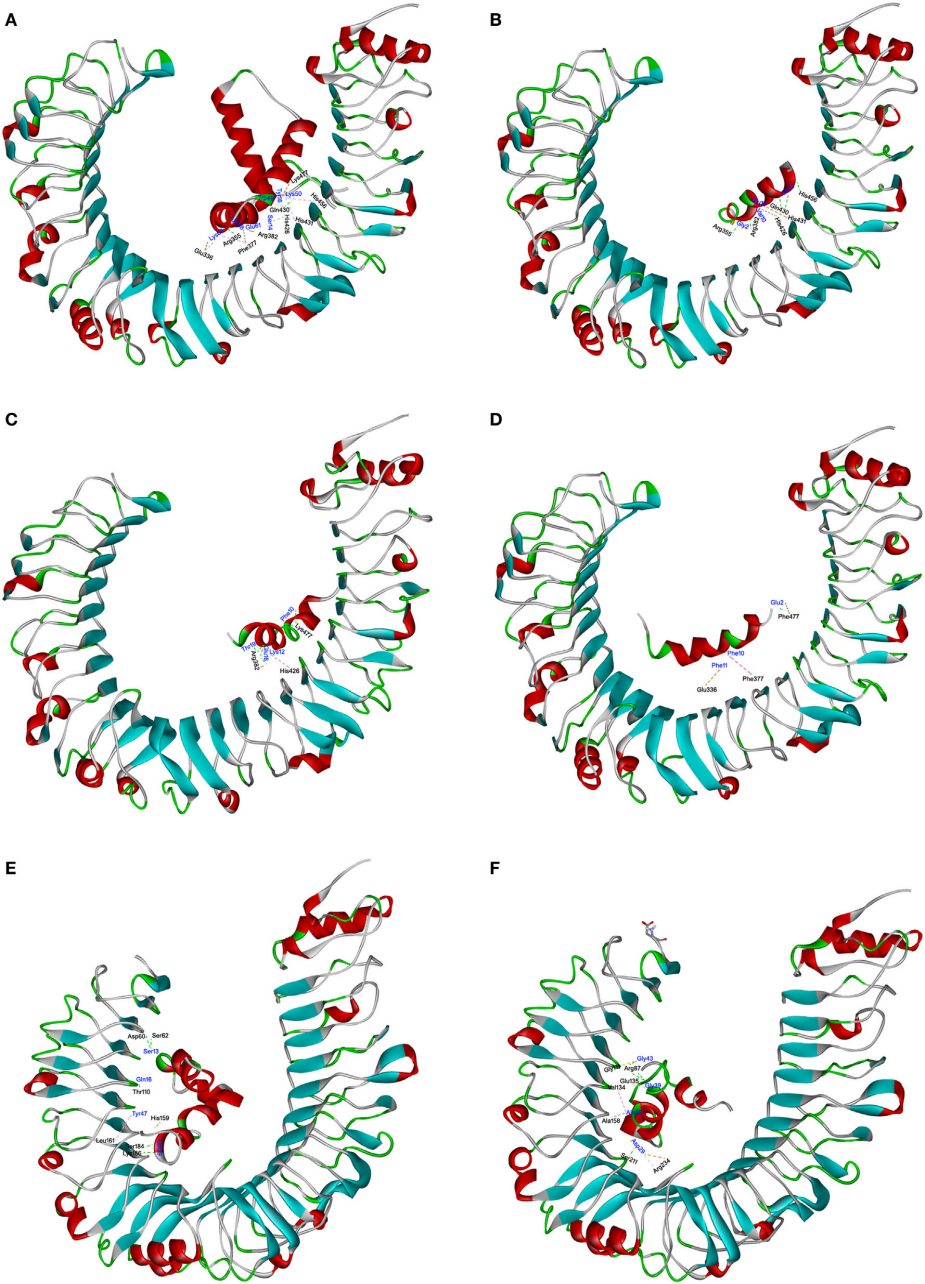
The interactions of toll-like receptor (TLR) 4 with HMGB1 and *Staphylococcus aureus* phenol-soluble modulins (PSMs). The molecular interactions of human TLR4 (hTLR4) with HMGB1 **(A)**, PSMα1 **(B)**, PSMα2 **(C)**, PSMα3 **(D)**, PSMβ1 **(E)**, and PSMβ2 **(F)** were shown as dashed lines with π–π, π–alkyl, and hydrogen bonds colored purple, pink, and green. The secondary structural elements were depicted as ribbons (coils, α-helices; arrows, β-sheets). Color was based on the secondary structures (α-helices, red; β-sheets, sky blue; loops, green). Residues from hTLR4 and ligands were labeled in black and blue, and shown as lines with carbon, oxygen, and nitrogen colored gray, red, and blue, respectively.

The interaction of TLR4 with HMGB1 and *S. aureus* PSMs was illustrated using molecular docking. The most plausible model of TLR4/HMGB1 complex was selected based on the details form previous literature about the binding site of HMGB1 on TLR4 (23, 39). The A box of HMGB1 (PDB ID: 2LY4) was docked into the pocket (336–477) of hTLR4 (PDB ID: 3FXI), thereby forming maximal van der Waals interaction with surrounding residues (Glu^336^, Phe^377^, His^426^, His^431^, His^456^, and Lys^477^) along with additional hydrogen bonds with Arg^355^, Arg^382^, and Gln^430^ (Figure 6A). Consequently, HMGB1 bound to TLR4 with a *K*_D_ value of 9.199 µM. The binding energy of the HMGB1–TLR4 complex was calculated using force field CHARMm which showed the potential energy to be −324.17 kcal/mol. Notably, molecular docking stimulation revealed that PSMα1 was bound into the same binding site as HMGB1 in hTLR4 through potential H-bond with Arg^355^, Arg^382^, Gln^430^, and His^456^, and multiple hydrophobic interaction with His^426^ and His^431^ (Figure 6B). PSMα2 was found to compete with HMGB1 for binding with Arg^382^, His^426^, and Lys^477^ (Figure 6C). And PSMα3 was shown to share interactions at Glu^336^, Phe^377^, and Lys^477^ with HMGB1 (Figure 6D). In addition, the docked complex of TLR4 with *S. aureus* PSMs α1–α3 were more structurally stable and energetically favorable than HMGB1, un-bond interaction energy reaching to −528.31, −495.60, and −682.74 kcal/mol, respectively. Further study indicated that *S. aureus* PSMβ1–β2 fully extended into hTLR4 without interacting directly with the residues (336–477) at the HMGB1 binding site (Figures 6E,F). To validate the stability of TLR4 with HMGB1 or *S. aureus* PSMs, we performed standardized MD stimulations using Discovery Studio 2017R2. As shown in Figure S3 in Supplementary Material, the most plausible models of TLR4 with HMGB1, PSMα1–α3, and PSMβ1–β2 were stable. However, the complexes of TLR4/PSMα4 and TLR4/δ-toxin were not stable, which is consistent with the prior SPR results, indicating that PSMα4 and δ-toxin lack TLR4-binding capacity (Figures S3D,G in Supplementary Material). Collectively, the differences in the amino acid distributions on the surface of each PSM appear to impact the TLR4 binding capacity.

## Discussion

*Staphylococcus aureus* has emerged as a major cause of community and hospital acquired infections. Immune defenses against *S. aureus* may be enhanced by local release of DAMPs such as HMGB1. HMGB1 can signal through a family of receptors, thereby functioning as a DAMP that alerts, recruits, and activates innate immune cells to produce a wide range of cytokines and chemokines. However, TLR4, which has been identified as the dominant inflammatory receptor for HMGB1, had no impact or very limited impact on the host response during staphylococcal infections. In this regard, we gained insights into the molecular mechanisms underlying the ability of *S. aureus* to dampen the HMGB1-induced TLR4 signaling by the novel virulence factor PSMs.

Phenol-soluble modulins are a recently discovered family of short, amphipathic, α-helical peptides in staphylococci (3). In *S. aureus*, PSMs can be grouped into four shorter α-type PSMs (~20 amino acids, PSMα1–α4), two longer β-type PSMs (~40 amino acids, PSMβ1–β2), and δ-toxin peptides (26 amino acids), whose genes are arranged in three gene clusters (4). Ordered aggregation of the monomeric PSMs into a proinflammatory complex plays important roles in the pathogenesis of *S. aureus* infections (9–13). The TLR2-stimulating capacities attributed to the complex of PSMs in initial studies paved the way for further investigation (40). While more recent analysis of PSMs-receptor interactions indicated that monomeric *S. aureus* PSMs do not activate TLR2 directly but are required for mobilizing lipoprotein, the TLR2 ligands, from staphylococcal cytoplasmic membrane (26). Subsequently, PSMs modulate the capacity of dendritic cells (DCs) to respond to TLR2 ligands, leading to a tolerogenic phenotype (30). Of note, the induction of tolerogenic DCs by *S. aureus* PSMs is not specific for TLR2 activation (28). *S. aureus* PSMs can also inhibit antigen uptake, maturation, and cytokine production of DCs activated by TLR4 (28). It is likely that TLR4 involves in the biological activities of *S. aureus* PSMs.

Therefore, we identified the interaction between TLR4 and all the monomeric *S. aureus* PSMs, including PSMα1–α4, PSMβ1–β2, and δ-toxin. As a result, PSMα1–α3 and PSMβ1–β2 were shown to bind to TLR4, whereas did not activate TLR4/NF-κB signaling. By contrast, PSMα1–α3 significantly inhibited the HMGB1-induced TLR4/NF-κB signaling pathway. To analyze the critical involvement of monomeric *S. aureus* PSMs in the HMGB1–TLR4 interaction, we ought to generate the 3D structures of all these peptides. Fortunately, the 3D structures of PSMα1, PSMα3, and PSMβ2 have been elucidated using NMR (35). The backbones of PSMα1 and PSMα3 are primarily α-helical, forming a single amphipathic helix. While PSMβ2 is comprised of three amphipathic α-helicals that fold to reveal a hydrophilic surface and create a hydrophobic core. With the NMR structures of PSMα1, PSMα3, and PSMβ2 available, we successfully generated the 3D model of PSMα2, PSMα4, PSMβ1, and δ-toxin with MODELLER, and further refined using CHARMm. Molecular docking simulation revealed that all the monomeric *S. aureus* PSMs except PSMα4 and δ-toxin fully extended into the active cavity of hTLR4, thereby forming maximal van der Waals interaction with the surrounding residues.

It is likely that the binding capability of PSMs to TLR4 may disrupt HMGB1–TLR4 interactions, as well as HMGB1/TLR4/NF-κB signaling pathway. Thus, we further analyzed the interaction between HMGB1 and TLR4. The B box of HMGB1, proposed to be the preferred binding site for MD-2, is exposed, leaving the A box to bind and bend TLR4 (23). According to the most stabilized pose of HMGB1–TLR4 complex predicted by molecular simulation, the A box of HMGB1 was located in the active cavities of TLR4 (336–477) with a redundancy of aromatic, aliphatic, and acidic residues. The side chains from residues provide hydrophobic and electronic interactions to aid in neutralization for the positive charge of HMGB1. Remarkably, PSMα1–α3 competes with HMGB1 for interacting with the surrounding residues of TLR4 domain. As a result, *S. aureus* PSMα1–α3 significantly attenuated the binding affinity of TLR4 with HMGB1, thereby inhibiting the HMGB1-induced NF-κB activation and proinflammatory cytokines production.

It is noted that the HMGB1-induced inflammation is not only mediated by TLR4 but also by RAGE and TLR2. Recent work showed that TLR4 is required for HMGB1-dependent activation of TNF-α and IL-6 release in macrophages, whereas RAGE and TLR2 are dispensable (25). To examine the involvement of RAGE and TLR2 in HMGB1 signals, we performed blocking experiments using neutralizing antibodies against RAGE and TLR2. As a result, we found that HMGB1-induced TNF-α and IL-6 release in THP-1 cells were inhibited by anti-TLR4 antibodies, but not by anti-TLR2 or RAGE antibodies, which is consistent with previous studies. More importantly, HMGB1-induced TNF-α and IL-6 release in THP-1 cells which were preincubated with *S. aureus* PSMα1–α3 decreased significantly, indicating that PSMα1–α3 have the same function as neutralizing antibody against hTLR4 by specifically targeting HMGB1–TLR4 interactions. Further studies are needed to determine the role of PSMs in HMGB1 signaling *via* receptors other than TLR4, such as RAGE and TLR2. Collectively, PSMα1–α3 can act as novel TLR4 antagonists, which may evolve as an immune evasion means used by *S. aureus* to subvert the immune defenses. Modulation of this process will lead to new therapeutic strategies against *S. aureus* infections.

## Author Contributions

MC and YW designed the experiments; MC, MYZ, CJ, XC, and LG performed the experiments; MC, MBZ, and ZC analyzed the results; MC and ZC wrote the manuscript; YW revised the manuscript. All authors read and approved the final manuscript.

## Conflict of Interest Statement

The authors declare that the research was conducted in the absence of any commercial or financial relationships that could be construed as a potential conflict of interest.

## Supplementary Material

Figure S1 The binding curves of TLR4 with *Staphylococcus aureus* PSMs and HMGB1. Surface plasmon resonance analysis was performed to assess HMGB1 and *S. aureus* PSMs binding to human TLR4 (coated on the chip), including PSMα1 **(A)**, PSMα2 **(B)**, PSMα3 **(C)**, PSMβ1 **(D)**, PSMβ2 **(E)**, and HMGB1 **(F)**. The binding curves and *K*_D_ (μM) were shown. Data are representative of three experiments.

Figure S2 TLR4 is required for HMGB1 signaling in THP-1 cells. THP-1 cells were preincubated with 20 µg/mL mouse IgG (as control) or neutralizing antibodies (20 µg/mL) against receptor for advanced glycation end products, TLR2, TLR4, or PSMα1 (5 µg/mL), PSMα2 (5 µg/mL), PSMα3 (0.5 µg/mL) for 30 min. **(A)** THP-1 cells were transfected with a NF-κB-dependent luciferase reporter. The NF-κB activity of THP-1 cells was measured prior to stimulation with HMGB1 using luciferase assay. **(B)** The NF-κB activity of the preincubated THP-1 cells challenged with HMGB1 (1.0 µg/mL). The values for the medium treated cells were arbitrarily expressed as 1.0. **(C)** The preincubated THP-1 cells were stimulated with 1.0 µg/mL HMGB1 for 12 h. The expression of human TNF-α and IL-6 was assessed by RT-PCR. **(D)** The preincubated THP-1 cells were stimulated with 1.0 µg/mL HMGB1 for 24 h. Supernatants were analyzed for human TNF-α and IL-6 by enzyme-linked immunosorbent assay. Data shown are mean ± SEM (*n* = 5). Significance was calculated by Student’s *t*-test. ****p* < 0.001.

Figure S3 Molecular dynamics (MD) of TLR4 with *Staphylococcus aureus* PSMs and HMGB1. The stability of TLR4 with PSMα1 **(A)**, PSMα2 **(B)**, PSMα3 **(C)**, PSMα4 **(D)**, PSMβ1 **(E)**, PSMβ2 **(F)**, δ-toxin **(G)**, and HMGB1 **(H)** was validated using a standardized MD protocol through Pipeline Pilot (PP) using the CHARMm component in Discovery Studio 2017R2.
