# Supplementary figures and images for: Staphylococcus aureus Phenol-Soluble Modulins α1–α3 Act as Novel Toll-Like Receptor (TLR) 4 Antagonists to Inhibit HMGB1/TLR4/NF-κB Signaling Pathway
Source: Front Immunol. 2018 Apr 25;9:862. doi: 10.3389/fimmu.2018.00862 (PMC5996891; doi:10.3389/fimmu.2018.00862)

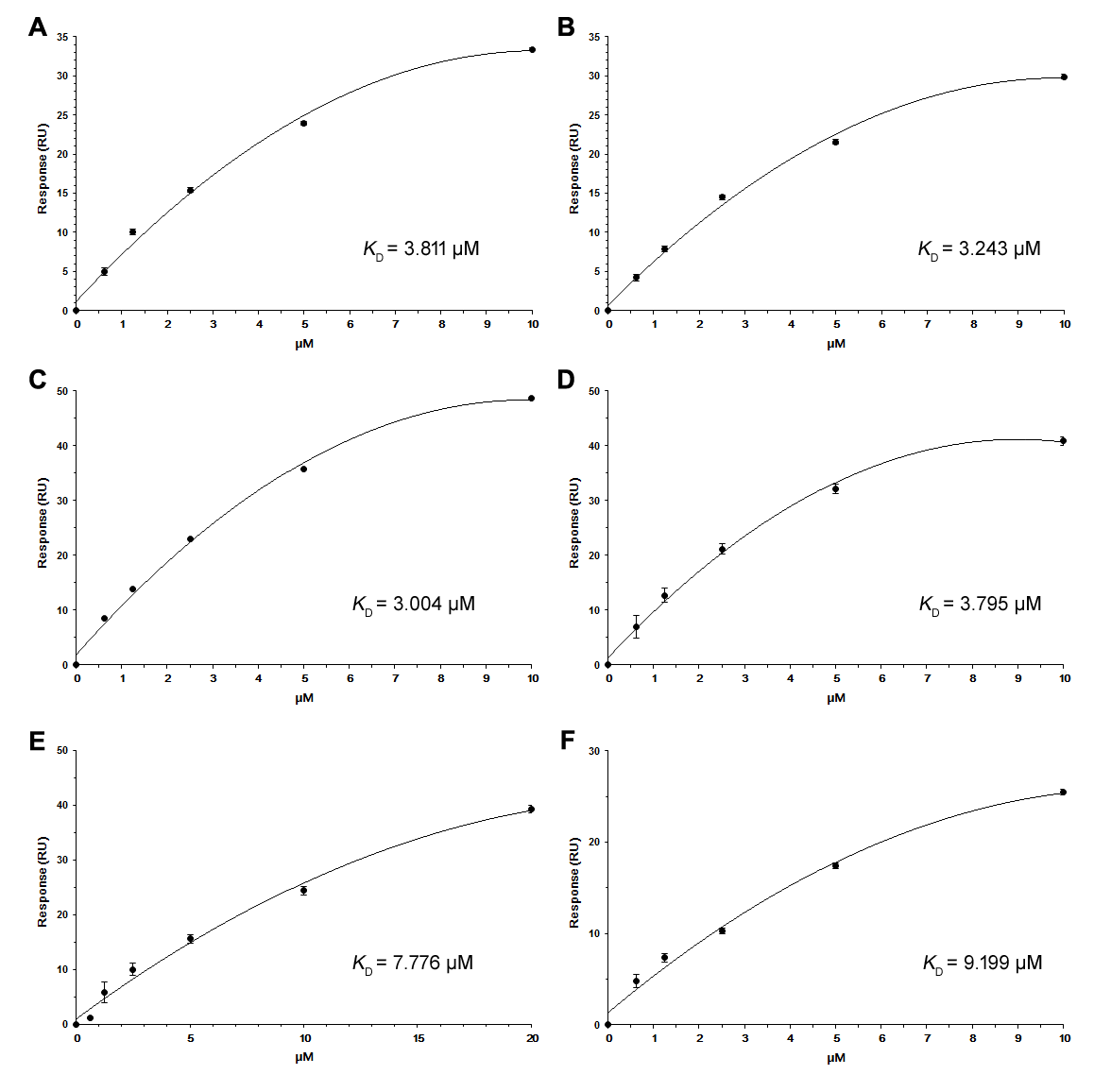

Supplement: Figure S1 — The binding curves of TLR4 with Staphylococcus aureus PSMs and HMGB1. Surface plasmon resonance analysis was performed to assess HMGB1 and S. aureus PSMs binding to human TLR4 (coated on the chip), including PSMα1 (A), PSMα2 (B), PSMα3 (C), PSMβ1 (D), PSMβ2 (E), and HMGB1 (F). The binding curves and KD (μM) were shown. Data are representative of three experiments. [file image_1.TIF]

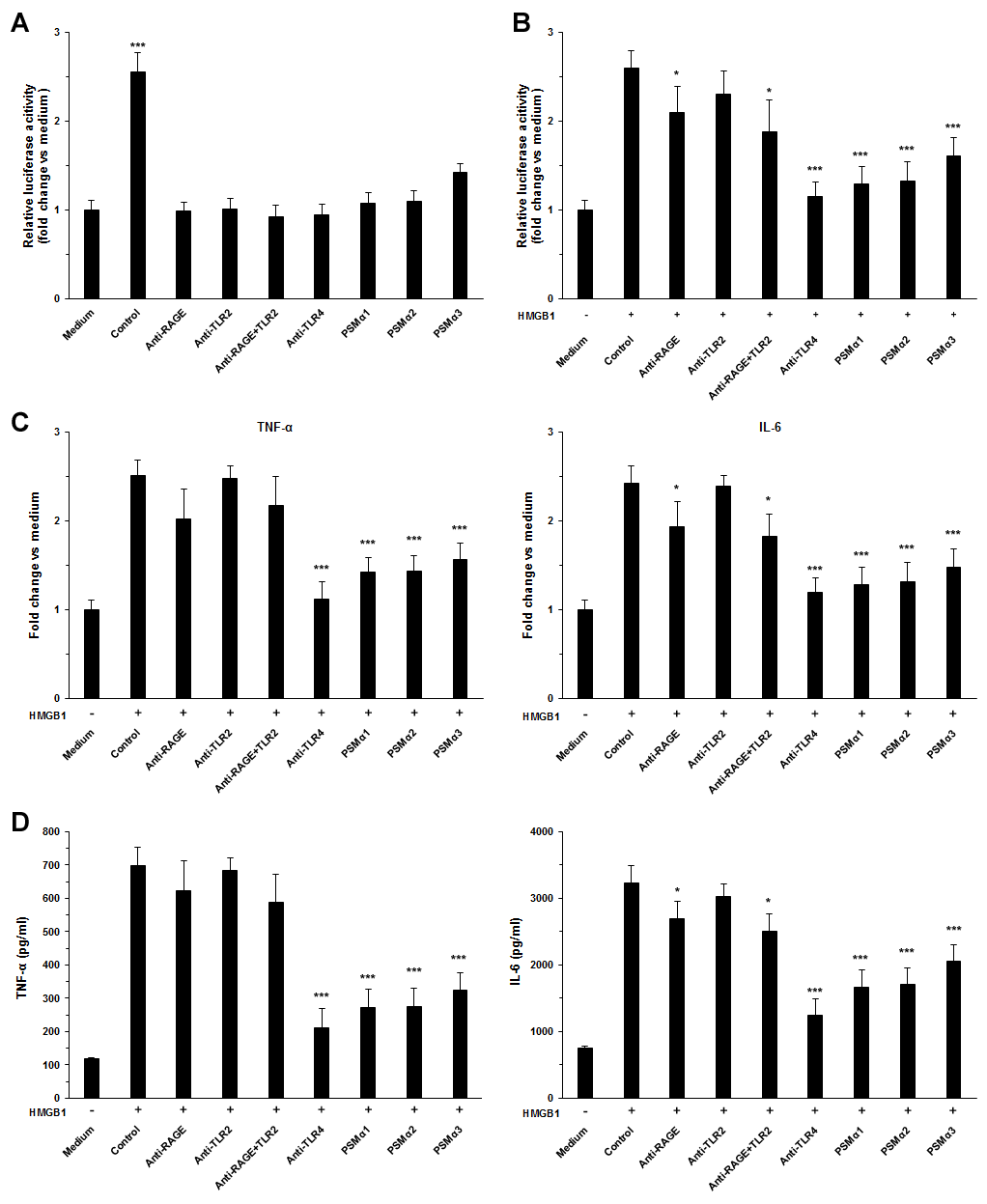

Supplement: Figure S2 — TLR4 is required for HMGB1 signaling in THP-1 cells. THP-1 cells were preincubated with 20 µg/mL mouse IgG (as control) or neutralizing antibodies (20 µg/mL) against receptor for advanced glycation end products, TLR2, TLR4, or PSMα1 (5 µg/mL), PSMα2 (5 µg/mL), PSMα3 (0.5 µg/mL) for 30 min. (A) THP-1 cells were transfected with a NF-κB-dependent luciferase reporter. The NF-κB activity of THP-1 cells was measured prior to stimulation with HMGB1 using luciferase assay. (B) The NF-κB activity of the preincubated THP-1 cells challenged with HMGB1 (1.0 µg/mL). The values for the medium treated cells were arbitrarily expressed as 1.0. (C) The preincubated THP-1 cells were stimulated with 1.0 µg/mL HMGB1 for 12 h. The expression of human TNF-α and IL-6 was assessed by RT-PCR. (D) The preincubated THP-1 cells were stimulated with 1.0 µg/mL HMGB1 for 24 h. Supernatants were analyzed for human TNF-α and IL-6 by enzyme-linked immunosorbent assay. Data shown are mean ± SEM (n = 5). Significance was calculated by Student’s t-test. ***p < 0.001. [file image_2.TIF]

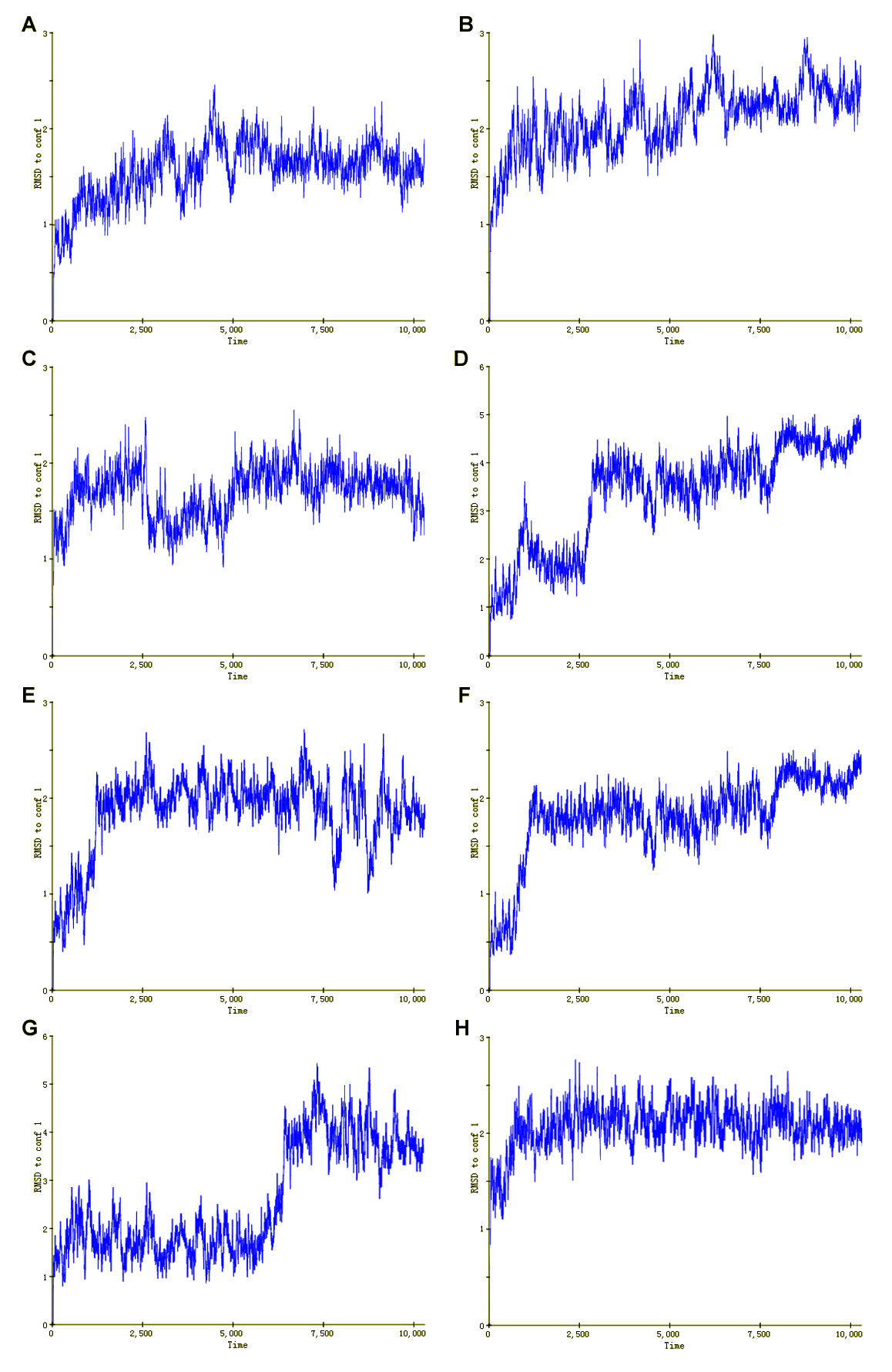

Supplement: Figure S3 — Molecular dynamics (MD) of TLR4 with Staphylococcus aureus PSMs and HMGB1. The stability of TLR4 with PSMα1 (A), PSMα2 (B), PSMα3 (C), PSMα4 (D), PSMβ1 (E), PSMβ2 (F), δ-toxin (G), and HMGB1 (H) was validated using a standardized MD protocol through Pipeline Pilot (PP) using the CHARMm component in Discovery Studio 2017R2. [file image_3.TIF]
